# On the Relations Which Dental Caries, Etc. Continued from Last Issue

**Published:** 1883-02

**Authors:** John R. Mummery


### THE DENTAL REGISTER.

Vol. XXXVII.] FEBRUARY, 1883. [No. 2.

### COMMUNICATIONS.

### On the Relations which Dental Caries,—as discovered amongst the Ancient Inhabitants of Britain, and amongst existing Aboriginal Races,—may be supposed to hold to their Food and Social Condition.

By John R. Mummery, Esq., L.D.S., F.L.S.

[Continued from January number.]

On the decline of the Roman Empire the enervated Britons were unable to withstand the ravages of the Picts and Scots, against whose entrance into England the wall of Hadrian now proved an insufficient barrier. They had destroyed every Roman town in the west, from the banks of the Solway to Gloucestershire, before the arrival of the Saxons; and I have explored the charred remains of the old city of Uriconium, in Shropshire, which was burnt by the Picts about A.D. 450. The Britons, having much Germanic blood among them, very naturally called in Teutonic allies, and amidst the distractions of the time, we can readily understand how those, who came ostensibly to help the Britons, remained permanently to occupy their land. Whilst the Jutes took possession of the country from Kent to Hampshire and the Isle of Wight, the Angles settled in Norfolk, Suffolk, and the north-eastern districts; and the Saxons in Essex, Middlesex and Wessex. These nearly allied races were of rude and barbarous habits, and too often, like the Pictish invaders, swept away the beneficial effects of Roman civilization as they advanced in their destructive course. Of this we have a notable example in the Romano-British city of Anderida (now Pevensey), the inhabitants of which were all slain by the Saxons, and the city laid in ruins. Reared amidst the Pagan superstitions of the North, they practiced cruel rites, very similar to those of the earlier British tribes. This is evident from the many instances in which the remains of those who were probably slaves or captives, are found buried in the graves of the chieftains during the earlier period of their settlement in England. Their teeth, as may be expected, exhibit signs of increased vigor of constitution; but I find a great difference in the teeth of skulls from various localities as regards both attrition and caries. This circumstance is probably due to such skulls belonging respectively to the earlier barbarous and the later civilized conditions of the Anglo-Saxon people, although their respective characters are not sufficiently distinct to make their recognition easy. In many of the earlier interments during their state of heathenism, the bodies were burned and the ashes preserved in urns, of much ruder form than those of the Romano-Britons; but so strong was the opposition of the Christian teachers to the practice of incremation, that it was abandoned about 150 years after the coming of Hengist and Horsa, in Kent and Anglia; although later in Mercia. The Anglo-Saxon bodies, simply buried, were laid in an extended position without coffins; thus differing both from the old British tribes, who placed the bodies in a contracted posture, and from the Romans, who usually laid them in coffins. Each northern wave of uncivilized people, when it reached England's shores, was an agent of fearful destruction; but in course of years a more peaceful time arrived, and therewith an advance in physical comfort and mental culture. Thus the subjugation of the Britons was followed by a commingling of their race with that of the Anglo-Saxons, and eventually the nation advanced in civilization,* When the Saxons arrived in Britain they found the fields and gardens well cultivated, upon the method introduced by the Romans; and after they had at

* I am informed by an eminent authority on these subjects, that the present agricultural population of Lincolnshire is made up of round-headed and oval-headed people, in about equal proportions, who are probably the descendants respectively of the Romano-Celtic and Anglo-Saxon races.

length obtained peaceful possession of the land, they so improved their farms by adapting their agriculture to the moist climate, that an abundance of wheat, barley, beans, and other crops were produced. Extensive herds of cattle and sheep were kept for the supply of the table, whilst the woods were occupied by multitudes of swine.*

A long list is enumerated of the domesticated fowls in a Saxon farm-yard, comprising, with few exceptions, all the species with which we are now familiar; whilst the deer, wild boar, and a profusion of feathered game supplied variety to the repast of the Saxon thane. They also made excellent beer, and imported wine from the continent, whilst the ladies drank mead, or other liquors prepared from fermented honey. Indeed, the wealthy Anglo-Saxon lived fully as well as any modern Englishman.

I am indebted to Professor Rolleston for my acquaintance with a very curious work, recently published under the direction of the Master of the Rolls, consisting of Anglo-Saxon manuscripts, most of them now published for the first time, accompanied by a translation. This remarkable collection of documents bears the quaint old English title of "Leechdoms, Wortcunning, and Starcraft," which, rendered into modern language, would signify "physicians' prescriptions, the knowledge of plants, and astrology," The book gives us a curious insight into the diet and customs, the diseases and remedies, which prevailed more than a thousand years ago in this country. In a monastic colloquy, or exercise-book for teaching students their own language and also Latin, a boy is asked to tell what he has to eat. He replies worts (that is, kitchen herbs), fish, cheese, butter, beans, and flesh meats. He drinks ale when he can get it, or, failing that, water.

In my examination of seventy-six Anglo-Saxon skulls, I found caries present in twelve cases, of which number it occurred in five instances on the approximal surfaces. In seven cases the molars had undergone severe attrition. In a large proportion of instances the teeth are very robust, of which a notable example is seen in

* In the Domesday Book of the Norman Conqueror we find the size of a wood estimated, on the several estates, by the number of hogs it is capable of maintaining on the acorns, beech-mast, and roots it produces.

the lower jaw of the celebrated chief, Brighthelm, now in the Hunterian Museum, the third lower molars being remarkably developed.

In five cases I found the third molars wanting, and also met with one case of irregularity, in which both the upper cuspidati are lying in an oblique direction on the palatal side of the dental arch, causing great protrusion of the lateral incisors.

The proportionate amount of caries is greatly diminished when compared with that found in Romano-British skulls, and we may reasonably assume that the simpler habits of the Anglo-Saxons, together with nourishing food, tended to maintain a higher standard of health, and consequently a better condition of the teeth than their predecessors enjoyed. I have, however, shown that they had some experience of toothache, and will now quote a few passages from the old documents to which I have referred, illustrating their ideas on the anatomy and pathology of the teeth, and the remedies they employed. First, as to the structure and diseases of the teeth.

"It is often inquired whether teeth be of bone, since every bone hath marrow, and these have no marrow; and other bones, though they may be broken, may by some leechcraft be healed, but the tooth never, if it be broken."

"The worst humor cometh to the teeth from the head; the moisture of the head falleth upon the teeth, and pierceth through them, causing them to rot and swell, so that the teeth can endure neither heat nor cold, and especially the grinder teeth, which are fastened, each with four roots,* and then they leave their roots, then they turn black and fall. This then is the leechcraft in that case: —

Take some part of the hide of a hart and a new crock, and water, and seethe three times as strongly as water boiling flesh-meat.

"Let the man hold this water in his mouth, as warm as he can bear it, till it be cooled. Then let him often take warmer, again casting it forth, and he will soon be mended."

* I cannot say that my examination of the skulls has supplied a single example of this abnormal number of roots. !

"The herb elecampane is good for soreness and wagging of teeth."

Leechdoms for sharp pain in the teeth, and for worms in the teeth, either for the upper or for the nether:—

"For tooth wark, bray to dust the rind of nut-tree and thorn-tree; cut the teeth on the outside, shed the dust on frequently.

"For the upper tooth-ache take leaves of withe wind, and wring them on the nose.

"For the nether tooth-ache, slit the gums with the instrument till they bleed."

"For tooth wark; if a worm eat the tooth, take an old holly-leaf, hartwort and sage. Boil in water, pour it into a bowl, and yawn over it; then the worms shall fall into the bowl."

"For tooth-worms take acorn-meal and henbane seed and wax, of all equally much. Mix them together. Make them into a wax candle, and burn it; let it reek into the mouth, put a black cloth under, then shall the worms fall on it."

It is a curious fact, illustrating the persistency of traditional remedies, that with some modification of the mode of burning the henbane seed, it is extensively used in many districts of England at the present day in the firm belief that the lobes of the exploded seed, which move slightly on bursting with the heat, are really worms expelled from the carious teeth.

In Dr. Wright's interesting work on the domestic manners of the Anglo-Saxons, a curious quotation is given from the laws relating to personal injuries, and the payment of compensation-money is proportioned with extraordinary minuteness. If any man struck off an ear he was to pay twelve shillings; for a nose cut through, nine shillings; for an eye, fifty shillings; for each of the four front teeth, six shillings; for the tooth which stands next to them, four shillings; and for that which follows, three shillings; for all the others, one shilling each. I am sorry that our ancestors did not better appreciate the relative value of their molars.

We have thus traced, with some degree of certainty, the history of dental caries from the times of the aborigines of Britain down to the settlement of the country under Anglo-Saxon rule, recognizing in each epoch, the influence of various modes of living on the condition of the teeth; and it was originally my intention to follow the subject throughout the centuries which have elapsed since the consolidation of the Anglo-Saxon kingdom, down to the present time. It would have been interesting to have examined the result of a fresh northern invasion by the vigorous and half-barbarian Danes, who, during the period of their dynasty, settled in many parts of England, as indicated by the termination of the names of such towns as Derby, Whitby, Grimsby, &c.

Again, the influence of the luxury introduced, through intercourse with the Normans during the later period of the Saxon rule, culminating with the Norman Conquest, and the establishment of the feudal system, would be well worth inquiring into.

Whilst the serfs were reduced to the hardest fare, the barons and their retainers were revelling in a profusion of luxuries; and most of my audience are familiar with that admirable illustration of the times given by Sir Walter Scott, in "Ivanhoe," in the colloquy between Wamba and Gurth, the swineherd.

I had hoped to recover traces of the great battles of Hastings, Shrewsbury, Bosworth, Naseby, and others; but, to my great surprise, I have not hitherto seen one satisfactorily authentic skull from the scene of the first-named decisive contest, and my explorations and inquiries respecting the other battle-fields have thus far been inconclusive; although I indulge the hope that I may yet attain to some accurate data relative thereto. Unfortunately, whilst explorers in past years have diligently collected coins and relics of armor or weapons, skulls have been till recently little valued, and have in too many instances been destroyed. Much uncertainty of date attaches to the great collections of human bones at Ripon, Rothwell, and other places, and the age of the celebrated Hythe skulls, so well described in the excellent paper by Mr. Cartwright and Mr. Coleman, is still a matter of debate among antiquaries.

I had also wished to ascertain, if possible, the effects upon the teeth, of that great change which occurred in this country, when tea, coffee, and other hot beverages were to a large extent subitituted for ale and wine; and I am not without hope that I may yet obtain some evidence on this subject.

We will now turn our attention to some of the existing uncivilized races of mankind; but the reasonable limits of a paper would be far exceeded if I availed myself of all the accumulated data which have resulted from my protracted inquiry. It will therefore suffice for our present purpose to make a selection of various tribes living in different climates, and subsisting on very diverse articles of food. For many years past, I have embraced every opportunity of obtaining the direct testimony of medical or other intelligent observers, who have resided amongst, or visited such peoples, and have found their evidence strikingly confirmed by the state of the teeth in the skulls examined of each respective race; and where this source of information has failed, I have consulted the works of trustworthy travelers, but there are still several groups of skulls which I am not yet in a position to utilize. A source of possible error occurs in the fact that skulls presented to public museums are usually selected with teeth in as perfect a condition as can be obtained, and having sometimes found a considerable difference in the proportion of caries, in various collections, I have as far as possible obtained a correct average.

It will be observed that I have avoided all reference to modern European and Anglo-American races, but I hope I may be able at some future time to obtain sufficient data to enable me to arrive at an approximate conclusion, and that our American brethren will lend their efficient aid in carrying out so desirable an object.

In order fully to appreciate the relation of food to healthy teeth, we shall do well to consider the influence which the various kinds of diet exert upon the human organization. These may be classified as nitrogenous or tissue-forming, carboniferous or heat-producing, and mineral (the last comprising lime, soda, potass, iron, &c.).

It should be borne in mind that oil, fat, sugar, gum, and similar articles of food,—with starch, whether obtained from grain or other vegetable sources,—only serve to maintain animal heat by uniting with oxygen in the process of respiration, and that any surplus, not so consumed, is stored up in the system as fat; but that no food, however rich in carbon alone, can either build up the tissues of the body or repair their waste, and that a man would starve if restricted to such food exclusively. Active exercise in cold climates, where the air is dense, accelerates atomic changes in the body; and thus a large supply of heat-producing food is needed to compensate for the rapid organic combustion.

In tropical countries, on the other hand, where the air is greatly rarified, a diminished supply of oxygen is inhaled, and less food is demanded, whilst those who consume much fat, butter, or sugar with their food, and take little exercise, become obese and indolent.

The man who undergoes severe bodily exercise in the open air takes more oxygen into his system, and requires more food, because the waste and molecular changes in the tissues of the body go on with greater rapidity, as the result of hard work; but it is a great mistake to suppose that severe mental exertion can be maintained without a corresponding supply of similar food. Close application to study induces waste of cerebral and nervous tissues, and those who have hard brain-work require an adequate supply of nourishing albuminous food to sustain their energies, equally with those whose pursuits require mere bodily exercise.

It appears to be a matter of secondary importance whether the nitrogenous elements of food, albumen, fibrine, and caseine, are derived from the animal or the vegetable kingdom. In the Arctic regions, where there is scanty vegetation, flesh and blubber supply the place of the grain, fruits, and roots of warmer regions, and health and vigor are maintained under either condition. Dr. Mantell has called fossils "Medals of Creation"; the teeth may be termed Medals of the Human Organization, inasmuch as their physical characters serve to record the pathological condition of the system at the particular age at which each class of teeth is formed, and, like medals, the only subsequent change they can undergo is deterioration. Under favorable circumstances, other parts of the animal frame may be strengthened, but the teeth have no such recuperative power, and must retain their originally defective condition during the period of their existence. Taking these facts into account, we may readily see that an ample supply of appropriate nutritious food during childhood is of greater importance in its effect on the development of healthy teeth than of any other structure of the body.

We now proceed to consider the extent to which these views are supported by a careful examination of the teeth of various races, living under the most opposite conditions with regard to climate, food, and habits.

The Esquimaux are stated to derive their name from an old Indian word signifying "eaters of raw flesh," and their favorite repasts fully justify the title. They are a littoral people, of the Mongolian type, inhabiting the coasts of North America from Labrador to Greenland and Behring's straits, and are generally nomadic in their habits, which will account for the great variety in their food and certain contrasts in the condition of their teeth.

With few exceptions, the vast region they inhabit lies far beyond the extremest limits of forest growth, and it includes the most desolate and inhospitable part of the globe. Although the land supplies comparatively little, the sea yields ample provision for all their wants, and they never live far from its shores.

In the summer they hunt the musk ox and reindeer, preserving the flesh under masses of rock and ice for future use; they have also an abundance of wild swans, geese, ducks, and sea-fowl at that season, with vast quantities of salmon and other fish in some localities.

The sea-bear and whale, the walrus and seal, supply the largest proportion of their food. Whilst cutting off the blubber from a whale they devour large slices of the raw flesh, eating as they work, and the whale's skin is esteemed an especial delicacy; but they prefer seal to all other flesh, drinking bowlfuls of the warm blood.

From ten to fourteen pounds of raw meat per diem is considered a reasonable allowance for each person. So short is the summer, that only a few stunted shrubs exist, and the fruit is so scanty that vegetable food cannot be reckoned as an element of any importance, excepting that in times of extreme scarcity the Esquimaux sustain life with the lichen which forms the food of the reindeer.

It is worthy of remark that Arctic explorers from a more civilized region, whatever disgust they may at first have experienced at Esquimaux food, have in many cases felt compelled by the low temperature to resort to a highly oleaginous diet, and have learned eventually to relish raw seal's flesh and blubber as thoroughly as the natives.

The people are short in stature, and of robust powerful frame. The jaws are singularly well-developed, and the teeth perfectly regular in position. The zygomatic arch is wide, and the tuberosity of the upper maxilla much prolonged. The teeth are often deeply worn on the grinding surfaces, frequently to the pulp-cavity, which gradually becomes obliterated by the formation of secondary dentine; and in one instance only among the sixty-nine skulls I examined, had alveolar abscess ensued. I found one case of caries of the first right lower molar on the grinding surface; in another case the first and second left lower molar had been removed: these were the only traces of disease.

In some skulls the first molars were affected much more than the other teeth, being worn obliquely towards the lingual side in the upper jaw, and towards the buccal side in the lower. As this condition of the teeth is observable in other races who live on hard food, it is probably attributable in this case to the mastication of tough sinewy raw flesh. In other Esquimaux skulls the teeth are worn down on an uniform plane, the front teeth being truncated like those of the Egyptians, a condition which is probably owing to the mixture of sand with the food in some localities.

The Indians of the north-western coast of America differ considerably from the Esquimaux, many of these tribes being known as "flat-heads," from their strange practice of distorting the cranial bones in early life by pressure-boards. Living in a lower latitude, they have not the same constant need of oily food, and they subsist chiefly on salmon, which are by far the most common of all fish in the rivers of the North Pacific. These fish ascend the rivers in such countless multitudes as to impede navigation, and during the prevalence of a south-west wind are driven ashore by thousands. The Indians split and dry the salmon on the sandy shore in a very rude and careless manner; and so large a quantity of sand adheres to this food, that their teeth are destructively and quite evenly worn down. They occasionally hunt the moose-deer and other animals, drying their flesh also for winter use.

Among fifty-one skulls I found but two cases of caries; in one instance two teeth were affected, in the other one tooth only. The teeth are frequently worn down to the margin of the enamel, but the dentinal pulp is invariably calcified; and among the fifty-one skulls examined I found the surprising number of thirty-two in which the enamel was entirely removed from the grinding surfaces of the molars and premolars, and from the cutting edges of the cuspidati and incisors.

The various tribes of North American Indians, who roam over the prairies and forests of the interior, are chiefly sustained by the chase of the musk-ox, moose, bison, and other large game; but no animal food comes amiss to them, and they eat the flesh of the beaver, fox, wolverine, and all the fur-bearing animals, whose skins they sell to the agents of the Hudson's Bay Company. With the occasional addition of a few roots, their diet is exclusively of an animal nature, but they are often subject to great privation. The maxillary arch is finely proportioned, and among twenty-one skulls of these tribes I found two cases of two carious teeth in each. A striking contrast is seen between the teeth of the fish-eating Indians and of the Indians of the interior, with respect to wearing down, only two cases being noticed of extreme attrition on the grinding surface, most probably explained by the absence of sandy admixture with their food.

Turning our attention to the warmer regions of South America, we find a people living between the twentieth and fortieth parallels of latitude, in the Argentine Republic, known as Gauchos. They are a mixed race of Indian and Spanish blood, who are employed at the ranches or great cattle-stations, and spend the greater part of their time on horseback, in hunting the half-wild cattle which roam over the wide grassy plains extending from the Atlantic coast to the foot of the Andes. I obtained some important information relative to their food and health from a gentleman who owns a large estate in Entre-Rios. These people live entirely on roast beef, with a little salt, scarcely ever tasting farinaceous or other vegetable food, and their sole beverage is Mate or Paraguay tea, taken without sugar. My informant assured me, that although his people always sought from him remedies for their various ailments, he never heard of more than one case of toothache. Indians of the same race inhabit the towns; but their habits are widely different, and their diet is a very mixed one, as they indulge greatly in Italian, French, and Spanish cookery, being fond of acid stews and made dishes. They eat immoderately of confectionery, and drink their maté with much sugar, indulging also in inferior wines. Caries in the teeth prevail extensively among these people, in striking contrast with the inhabitants of the open country. I may add that these statements are confirmed by the observations of a naval medical officer who was stationed some time in the Rio de la Plata.

I have been unable to see a sufficient number of skulls from this district to furnish reliable evidence.

We have another example of a race subsisting entirely on animal food, in the Arabs who inhabit the Nubian Desert, a district which consists principally of hills varying from 1,000 to 1,800 feet high, and is destitute of all vegetable products suitable for human food. Their camels subsist on the thorny shrubs growing among the rocks; and the milk and flesh of these animals (with salt) constitute their sole ordinary food.

On their occasional journeys into Egypt to sell camels, they usually bring home a small quantity of wheat, which is never ground, but boiled into a kind of frumenty and eaten as a luxury; but it must not be reckoned as an ordinary element in their diet.

I learn from Mr. Bonomi, who had abundant opportunity of studying the habits of these people, that they have most beautifully regular and sound teeth, and that they constantly clean them before and after every meal—an infrequent practice, among the ruder tribes of mankind.

These statements are confirmed by Dr. Murie, who saw much of these people during his journey along the course of the Nile, and particularly observed the sound state of their teeth; and the same may be said of the few skulls I have seen. I am informed also, by my friend Mr. Lockhart,—who established the hospitals at Shanghai and Pekin, and had ample opportunities for observing the peculiar diseases of the various tribes in the Chinese empire,—that he had particularly noticed the fine teeth of the nomadic people from Chinese Tartary. These tribes roam over the extensive grassy plains, rearing large herds of oxen and sheep, and are a tall, fine race of men; they have no vegetable food, but live entirely on mutton. They bring their cattle to Pekin for sale, and on these occasions have often sought medical aid for ophthalmic and other complaints, but never for affections of the teeth; which, Mr. Lockhart observed, were always sound, and regular in position.

Although, through the influence of Christianity and civilization, the practice of cannibalism is happily becoming gradually discontinued, I must not omit to recognize its extensive prevalence only a few years since, comprising the period during which the skulls I examined were collected. This revolting custom exists under very different conditions.

The Fans of Western Africa devour the flesh of enemies slain in battle; but it is also said, on good authority, that the inhabitants of one village are in the habit of purchasing the bodies of those who have died naturally in a neighboring one—making payments in ivory.

The miserable inhabitants of Tierra del Fuego are addicted to the barbarous practice of cannibalism when at war; and it is affirmed that when pressed in winter by hunger, they kill and devour their aged women--alleging, as an excuse, that they cannot catch fish or game.

The Australian, under similar circumstances of extreme destitution, is known to club and eat his child, wife, or neighbor, but the Fejee Islanders and New Zealanders have no such excuse,--living, as they do, in countries which always supply sufficient food to prevent a famine—but are murderous cannibals who prefer human flesh to every other kind of food. The habits of the Fejees are minutely described, so recently as 1858, by a resident Englishman, Mr Williams, from whose work I have derived my information.

Referring to the remaining cannibal tribes, he says that they slaughter all shipwrecked foreigners, or inhabitants of neighboring islands, who are cast on their inhospitable shores; and so far from regarding it as a crime, they boast on going to war, that they shall eat dead men's flesh to repletion.

Those who die a natural death are never eaten; but cannibalism is an element thoroughly interwoven with their social institutions.

If the people of a village give the most trivial offence to a powerful chief, he orders them to be attacked and killed. The bodies are brought home in triumph, and borne from the boats by women; or they invite their neighbors to friendly barter, when they are surrounded, killed, and baked in ovens. They are a tall, robust race of men; and among thirty-eight skulls I found only two instances of dental caries, one case in which two teeth were affected, and one of extensive disease.

The New Zealanders have too much resembled these people in their habits. They are a vigorous, energetic race, tall and muscular, and superior in intelligence to the people of Eastern Polyne-ia.

The love of war is with them the spring of every action and their great object in life, and until recently brought under the humanizing influence of Christian civilization, their country was the very center of deliberate murder and cannibalism; for not only their enemies, but unfortunate shipwrecked sailors, were almost invariably killed and eaten.

The country produces quantities of nutritive roots, those of the tree ferns alone supplying a sure resource in times of scarcity, and the shores abound in shell-fish, which are extensively eaten; an aliment which probably accounts for the unusual proportion of cases in which the teeth have suffered from severe attrition.

The maxillary arch is remarkably expanded, and the teeth are beautifully regular; among sixty-six skulls, I met with but two cases of caries, one in which two teeth, and in the other four, were affected.

On reviewing this catalogue of flesh-eating tribes, including true cannibals, and observing the generally sound condition of their teeth, it must be confessed, that an impression anything but agreeable is left on the mind; and it is with a feeling of relief that we can turn our attention to races, with whom the flesh of animals forms but one element in a more varied diet, or to other tribes who even abstain from it altogether, and yet enjoy, in many instances, a healthy condition of the teeth.

The inhabitants of Eastern Polynesia appear to great advantage with regard to their habits, when compared with the cannibals of the Western islands. They are usually above the middle stature, with well-formed limbs, and remarkably graceful figure, although inferior to the New Zealanders and Fejeeans in muscular development and physical power. The scenery of these islands is of the most magnificent character, and the vegetation so richly beautiful that voyagers have often styled the country a paradise. The people are very neat and cleanly in their habits, and spend much time in bathing; but their fine climate yields such an abundance of vegetable products, that they are somewhat prone to idleness, although capable, when necessary, of great exertion.

The bread-fruit, plantain, cocoa-nut, with many other fruits, and sugar cane, grow in profusion; but their usual food consists of plantains, beaten into a paste with cocoa-nut milk, and is called Popoé; they also consume a considerable quantity of fish.

The sea yields a good supply of turtle, and the people rear swine and poultry; but these articles of food are either reserved for the use of the chiefs or employed in trading with foreign ships. Among seventy skulls I found eight cases of caries, a large proportion extensive; and absorption of the alveolar margins was frequent. In nine cases two or more of the third molars were absent.

The people of the Sandwich Islands, lying to the north of the equator, in the corresponding latitude to that of the Society Islands on the south of the line, are less favored with respect to the natural products of their country. The soil is poor, and there are few wild animals or fish. The cocoa-nut, bread-fruit, and plantain are tolerably abundant; and oranges, lemons, grapes, and water-melons have been introduced; also goats, swine, cattle, and poultry; but the staple food of the people is the root of an arum, called taro, which is roasted and eaten as a substitute for bread;—to this we may add the sweet potato, sugar cane, and yam.

The inhabitants of these islands are of less graceful form and feature than those of the Southern Pacific Islands. They are of moderate stature; the face is broad and flat, the features are coarse, the muscles flabby, and there is a great disposition to obesity. Many Americans, and other civilized people, have settled in these islands, and the native race appears to be rapidly diminishing in numbers.

In twenty-one skulls four cases of caries occurred, two of which were very extensive, and I observed one case of that pitted state of the enamel of the incisors, canines, and first molars, usually known as honeycombed teeth.

The natives of that extended tract of country, Chili, lying to the west of the Andes, are a singularly industrious, active race. Like the Gauchos, on the Atlantic side of the great mountain-range, they are a mixed race of Indian and Spanish extraction, but their diet and occupations are very dissimilar. Their country is traversed in many districts by mountainous spurs, and consequently a great diversity of soil and climate exists: the seasons are as regular as in Europe, and the air is most exhilarating.

The principal native fruit is the kernel of the Araucarian pine; but the Chilian is a diligent agriculturist, and large quantities of excellent maize and wheat are produced, also pease, beans, and other leguminous seeds. Figs, grapes, and peaches are abundant. A striking contrast is observable between the Gauchos of the Atlantic and these Gausos of the Pacific flanks of the Andes. The former live exclusively on *animal* food, the latter almost as constantly on *vegetable* diet. The former are a pastoral, the latter an agricultural people; and their usual food is wheaten or maize bread, beans, and fruit.

The grass is very luxuriant, and in some districts cattle are reared, their flesh being dried in strips, chiefly for exportation, under the name of "charqui," or jerked beef. These particulars I obtained from a gentleman long resident in the country.

The extensive mines of Chili yield gold, silver, copper, lead, and tin, and the whole produce is brought to the surface by the personal efforts of the miners, who ascend shafts of 400 or 500 ft., carrying 200 lb, weight of ore on the back, with no other aid than that afforded by notched trunks of trees placed in zigzag.

These miners are healthy, cheerful, and active, yet their food consists chiefly of boiled beans, with the addition of bread and figs, and once a week, a little jerked beef. Among nineteen skulls,

I found two cases of slight disease in the teeth, and one in which five teeth were affected. The dental arch is finely expanded, and I observed no other signs of disease. It has often been remarked with respect to these Indian half-breeds, both of the flesh-eating Gauchos and the grain-eating Chilians, that their jaws serve the purpose of an additional hand, as they often pull up the saddle-girths of a restive half-wild horse by the aid of their powerful teeth, or by the same means hold the lasso, by which the wild cattle are secured.

The Australian races exhibit in the configuration of the skull the lowest type of the human family. They are for the most part improvident savages, and spend their lives in alternate feasting and famine.

During some portions of the year they hunt the kangaroo, opossum, and emu. Wild turkeys, geese, and swans often abound, and there are many varieties of fish on the coast. But they lay up no store, and when the game lies close in the winter rains, they resort to any food that can be obtained; as lizards, grubs, ants, frogs, snakes, or the roots of water-plants; and even, as already noticed, when driven by hunger, sometimes sacrifice the weaker members of the community to their cannibal appetite. The cranium in these people is very narrow, the forehead low and receding, the teeth, especially the lower canines, project in a striking manner. The jaw-bones are powerfully developed, and large in proportion to the cranium. The molar teeth are often of extraordinary size, especially the third lower molar, in one instance measuring five-eighths of an inch in width, and the enamel has a curiously porcelain-like appearance. In a considerable proportion of cases these organs are destructively worn, even beyond the limits of the enamel, exposing the pulp-cavity, and causing alveolar abscess. Irregularity of the teeth is not infrequent, and caries on the proximate surfaces often occurs; indeed, I have met with every form of dental disease with which we are familiar among the English race.

Among 132 Australian skulls I found caries present in twenty-seven cases, often very extensive.

The physical character and habits of the Tasmanian race (now nearly extinct) so closely resemble the Australian that I need only observe that in all points relating to disease, irregularity, or destructive wearing of the teeth, the defects of the Australian are exaggerated in the Tasmanian. In thirty-three skulls caries were found in nine cases, and in the majority, a considerable number of the teeth were affected.

The wide-spread African races next demand our attention, and I have collected a great amount of evidence with regard to the state of their teeth, having tabulated their condition in 438 cases. So remarkable a contrast is presented by the teeth of the multitudinous African tribes, that I have taken some pains to trace the cause of this difference in their condition, and to distinguish the several races. The Ashantees, Dahomeans, and some neighboring tribes on the West Coast are athletic, robust, and warlike ; they frequently overwhelm the provinces inhabited by feebler tribes killing great numbers of the people and carrying off the remainder as slaves.

Although they practice the diabolical custom of immolating their prisoners, as a part of the funeral rites of their chiefs, they do not appear to be guilty of cannibalism.

The country abounds with the elephant, rhinoceros, and hippopotamus, also with vast heards of deer, and many species of antelope, and the natives extensively cultivate the bananna and other fruits, sugar cane, yam, beans, pease, and maize. They have therefore no lack of nourishing food, and their bodily frame is powerfully developed. The maxillary arch is very expanded in form, and the frequency of supplemental teeth is a curiously interesting fact, as illustrated by the following instances :—

Case 1.—A fourth well-formed molar is present on each side of the upper jaw, the teeth occupying an exactly regular position in the dental arch.

Case 2.—A fourth upper molar is present on each side in the upper jaw, the supplemental teeth inclining slightly toward the buccal side of the arch.

Case 3.—A perfect fourth molar is present on the left side only.

Case 4.—A mal-formed fourth molar on each side.

Case 5.—A similar example.

Case 6.—Also resembling Case 4.

Case 7.—A well-formed right upper supplementary first bicuspid, occupying a natural position in the arch.

Case 8.—A supplementary left upper bicuspid in regular position.

Case 9.—A third upper right bicuspid on palatal side of maxillary arch. Caries have resulted in this case on the approximal surface of the additional tooth, also, on the second bicuspid.

Case 10.—A supplementary upper cuspid on each side, regular position.

Case 11.—A duplicate well-formed right upper central incisor.

It is a remarkable circumstance that I met with no example of a supplementary tooth in the lower jaw; but the third lower molar is frequently larger than the second.

I had the opportunity of examining the skulls of three of those noted female warriors, the Dahomean Amazons. The skulls were by no means remarkable for powerful development of the facial bones. In two cases the teeth were perfectly sound; in the third case the first and second molars on both sides of the upper jaw were slightly carious on their approximal surfaces.

In the Zulu Caffres, dwelling on the southern coast of Africa, we find a similar example of a race who have long been the terror of the neighboring tribes; and I have obtained a very full account of these people from a gentleman long a resident at Durba, in Natal. The Zulus are very superior in intelligence to all other South Africans, and are physically a powerful and athletic race. They have pursued their devastating course, conquering tribe after tribe, and killing or carrying away the inhabitants as slaves until a district 400 miles in length, which a century ago was thickly populated, became as desolate and barren as in its original state of wild bush, and all traces of human habitation were obliterated; the miserable remnant of the conquered people wandering without huts, cattle, or corn, and sustaining a precarious existence upon roots and shell-fish.

The Caffre country is well stocked with elephants, hippopotami, antelopes, deer, and wild boars. The manatee frequents the coast, waterfowl and fish abound, and they have extensive herds of cattle; but their ordinary food consists of curdled milk, millet, beans, and maze. The flesh of animals is a much-prized luxury, only consumed on special occasions, as in training of their warriors, who, for a long time before an intended foray, are supported almost entirely on the flesh of cattle, to give them strength and courage; a system of diet which is also recognized among civilized nations as that most conductive to the development of muscular power. In forty-nine skulls I find seven instances of caries, five of which were of very limited extent. The maxillary arch is of singularly fine proportions, and the teeth are usually very regular and perfect. It is worthy of notice that they make a constant practice of diligently cleaning the teeth after eating.

If we turn our attention to the more feeble African tribes from which the slave-markets are supplied, we see great deterioration in the state of the teeth; and, by a curious coincidence, the percentage of subjects in whom caries occurs is precisely the same on the eastern and western coasts, although the disease is far more severe in *degree* among the western tribes. These races inhabit marshy districts along the course of the great rivers, and are subject to fevers, rheumatism, and neuralgic affections; the general tone of health is often very low.

The plantain appears to be their staple food in times of peace; but from their frequent state of disquiet, owing to the marauding practices of the slave-hunting tribes, they often neglect cultivation, and are also deprived of their cattle. Under these circumstances they sustain life upon wild berries and roots; but this kind of vegetable food is found to be so insufficient to satisfy the demands of the system, that they have a special word, "Gouamba," signifying an irresistable craving for flesh-meat. If they are not so fortunate as to secure any of the larger animals, as the elephant, deer, antelope, etc., they are compelled to resort to the flesh of the monkey, rat, snake, or frog; but that of the dog appears to be preferred to every other.

I have examined no less than 268 skulls of Africans who have been brought down as slaves to the various points of the coast, and have died of disease or exhaustion; they vary so much in form, capacity, and thickness, that they evidently belong to many various tribes of the interior. The enamel of the teeth is sometimes very coarse in texture; in others very thin, fragile, and opaque, and the configuration of the dental arch often presents a striking contrast to its development among the more powerful Ashantees and Caffres. Of sixty-six cases of caries I found no less than thirty-five in which it occurred on the approximal surfaces, a condition evidently due to insufficient expansion of the maxillary arch, as a consequence of defective nutrition. In no less than sixteen cases the whole of the molars and bicuspides were carious, and (which is of rare occurrence among savage tribes) the incisors and canines were often involved. A widely-extended custom prevails of knocking out the right central incisor in early manhood, and I have found many instances in which, judging from the form of the jawbone, the molar teeth had been extracted, and not lost by a natural process of absorption.

The third molar in these tribes also is frequently larger than the second, and I observed among them five cases of irregularity from contracted arch, and many examples of misshapen molars and bicuspides, also four cases of supernumerary teeth, usually of distorted form.

The Bushmen are people of singularly dwarfish stature, distinct from the true negroes, and live in the deserts of Southern Africa from choice, possessing an intense love of liberty. They are the only real nomads in the country, never cultivating the soil or rearing any domestic animal but the dog, and are so intimately acquainted with the habits of the various species of antelope and other game, that they follow them in their migrations from place to place, deriving their chief subsistance from their flesh.

Notwithstanding the great skill of the Bushmen in the use of the bow and arrow, by which they secure feathered and other game, they are often reduced to the verge of famine by the protracted drought to which this country is liable, driving the wild animals away from their accustomed haunts.

Under these circumstances they eat locusts, caterpillars, and a species of frog, said by Dr Livingston to be nearly as large as a chicken. When these resources fail, they collect wild beans and edible roots, the most valuable of which are those of the succulent Mesembryanthemum family. Growing in these arid deserts, they are furnished with thick, fleshy leaves, having pores capable of absorbing moisture with avidity, retaining it in the driest atmosphere; and the large tuberous root often supplies by its succulent properties the lack of water.

As we might reasonably expect, the frequent privation of food produces its usual effect on the condition of the teeth. Among the twenty-nine skulls I have examined I have found six cases of caries, the majority being of an extensive character, and in nine cases the teeth had undergone destructive attrition.

The Hindoos residing in the southern parts of India are extensive consumers of rice, and it is usually spoken of as their staff of life. It is, however, a mistake to suppose that vigorous health and strength can be maintained on a diet so poor in flesh-forming material, and I have perused with much interest the elaborate official reports upon the diet of the inhabitants of every part of India. From these sources of information I learn that, although rice is the staple article of food throughout wide districts, there are no absolute vegetarians in India. Those who, like the strict Brahmins, abstain from flesh, consume much milk, curds, ghee (or clarified butter), fruits and vegetables. The Casters who are less strict in their dietary, on religious grounds, are accustomed to eat mutton, poultry, pork, goats' flesh, venison, or other nutritious meats, when their circumstances enable them so to do. The poorer classes supplement the deficient nutritive value of the race by millet and various kinds of dhall, under which designation are included lentils, vetches, beans, and other leguminous seeds. This kind of diet is, however, often indigestible if taken in large quantities; and when they can obtain fish they eat it, although very frequently in a semi-putrid condition. In various parts of the country they consume the flesh of the horse, the porcupine, squirrel, iguana, and frogs; also marine, fresh-water, or land snails. Indeed, no beast, bird, reptile, or fish appears to be unacceptable among some sections of the community.

The Mussulmen eat every kind of meat but pork, and the wealthier among them often prefer wheat (which is brought from the northern districts) to rice. A great variety of fruits, as the plantain, date, cocoa-nut, mango, etc., enter into their diet, and they use quantities of fresh vegetables, with hot condiments in their curries.

All classes manifest an extraordinary fondness for sweetmeats, which are supplied in endless variety and profusion. They are made of flour, sugar, and spice, and are boiled in ghee or syrup, old men eating them with as much avidity as children. An Indian officer informed me that a native cavalry regiment, known as Skinners' Irregular Horse, were never so well pleased with any reward for their services as with a supply of sweetmeats. After a victory, a regimental order was usually issued, directing that two pounds of sweetmeats should be served out to each man, the whole of which was consumed before the morning.

Among seventy-one skulls examined from Southern India, I found ten cases of caries; in three of which six teeth were involved.

If we turn our attention to the lofty temperate regions of Northern India, comprising the upper water-shed of the Ganges and Bramah-pootra, we find much greater simplicity in the diet of the inhabitants than is prevalent among those of the southern provinces.

An officer of the Royal Engineers, who was stationed for a considerable time in the district, informs me that their diet consists of unleavened bread made from wheat-flour, ground in hand-mills and not sifted (this is made into small cakes, called chupatties, baked, and eaten with salt and butter), which, with the addition of milk and cheese, and occasionally of boiled lentiles, constitutes their entire aliment.

The district is inhabited by three distinct races of people. The Kumalies were originally Thibetians, a pure Mongolian race; the Rohillas are of Affghan origin, and the Goojery Hindoos are of the same stock as those of the plains.

In many localities these three tribes exist side by side, preserving their distinctions of race; in others every variety of modification results from intermarriage; but in each case their diet is the same throughout the district.

My informant, who was stationed at a distance from any medical man, was frequently applied to by the natives for medicine to relieve their various disorders, but he never saw or heard of a single instance of toothache; and he particularly noticed that their teeth were very regular, of good color, only moderately worn down, and were frequently retained to an advanced age.

The Government official reports, referring to the people of this district, designate them a finely-developed race, muscular, strong, and active, far superior to the rice-eating inhabitants of the plains, and making much better soldiers.

We have here evidence that different races living under the same conditions are all alike influenced by them; and if we take the teeth as a test of the difference between the northern and southern people, we shall also find these statements corroborated.

In 152 skulls from Northern India I found but nine cases of caries, and in no instance were more than two teeth affected, presenting a very favorable contrast to those of Southern India, in which the numerical proportion of cases is more than double, and the extent of disease far greater in many instances.

I received from Count Wellowicz, a military surgeon in the British service, an interesting illustration of the effect of a similar diet upon the people in Abyssinia, living between Zoola and Senafe. They live principally upon the highly nitrogenized seeds of Holcus Sorghum, a kind of millet, and the Count assures me that he had ascertained that their teeth were perfectly free from decay.

The Maylays inhabiting the Indian Archipelago enjoy a luxurious climate, amidst tropical vegetation. Banannas, cocoa-nuts, and other fruits abound; birds and fish are also plentiful.

The people are, on the whole, a vigorous, healthy race, much occupied in maritime affairs, and too frequently addicted to piracy.

It is a common practice with these people to file the labial surfaces of the upper incisors and cuspidati deeply into the dentine, in some cases exposing the pulp-cavity, and giving rise to alveolar abscess. Among twenty-four skulls I found two cases in which two teeth were affected, and only one of extensive caries, which shows some improvement as compared with the teeth of the inhabitants of Southern India.

The Chinese live to a great extent under conditions as artificial as those of the inhabitants of European cities. I have received much valuable information from my friend Mr. Lockhart, who, when superintending the hospital at Pekin, had every opportunity of acquainting himself with the habits of the Chinese in every grade of society, that their diet is often very complex and unwholesome. Rice is their staple food; but in some districts they partially substitute millet.

They also eat quanties of salted cabbage in a semi-putrid condition, and prepare other vegetables in a similar manner, afterward frying them in oil. Those who can afford it eat pork, ducks, fowls, fish, and other animal food, which is cut into pieces and stewed in oil or soup.

All classes take their food quite hot, they never drink cold water, but they drink tea at all their meals, and also at intervals through the day. The beverage is invariably presented to visitors, and is sipped at first nearly boiling hot, the remainder being allowed to become lukewarm.

Their teeth are very faulty, and the incisors are often carious in early life; they are very neglectful in cleanliness with regard to these organs. The Manchoo Tartars, the inhabitants of Pekin, suffer equally with those of southern China.

Mr. Lockhart has also supplied me with an other example of the influence of differing physical conditions on the general health and that of the teeth in people of the same race.

The Chinese who inhabit the hilly country comprised in the Shangtung promontory, are employed by the Government to fetch the tribute of rice from the southern provinces. The grain is conveyed in canal-boats, and the voyage, which is very protracted, involves much physical exertion. These people are tall, their stature being rarely under six feet; they are very healthy, and possess singularly fine teeth. The opposite promontory of Corea is a marshy unhealthy district; the people are a small, stunted, and shriveled race, far inferior to the Chinese generally; they have miserable health, and suffer much from carious teeth. I

have examined twenty-seven Chinese skulls, in ten of which disease was present, and in eight of the number disease was very extensive.

That a predisposition to caries in the teeth, like other diseases, is largely dependent on general hygienic conditions, is, I consider, supported by evidence derived from a people much easier of access to us than the examples already quoted. In the picturesque region of the Italian Alps, extending from the eastern side of the "Vai Sesia" to the "Val Bregalia," a custom prevails strangely at variance with our English standard of propriety, and the practice has extended in some cases into the Canton Valais, in Switzerland. After the body of a deceased relative has been buried for a period varying from one to three years, the head is disinterred, and, having been duly cleansed by persons who cultivate that speciality, the name of the deceased is inscribed in the frontal bone, and the skull is placed in the mortuary chapel of the village—the cranium of a priest being distinguished by the black sacerdotal cap of the late owner.

Our feeling may revolt from the practice, as a breach of good taste and reverential regard for the memory of those whom we have loved and lost, nevertheless it affords an unusual facility for examining a large number of skulls in excellent condition; but the task is attained with some difficulty, and possible risk to the too curious explorer, as these ossuaries are provided with an iron grating, which is usually locked, rendering it a difficult matter to obtain accurate statistics. I have, however, availed myself of the opportunity afforded by four autumn holidays to examine the skulls, when I could gain admission without attracting notice; and I was struck with the singularly perfect condition of the teeth in nearly every skull, only a small percentage exhibiting any sign of caries; I found the dental arch well expanded, and the teeth regular, meeting but one solitary case of contracted arch, which was a thoroughly V-shaped upper jaw. The people inhabiting these elevated villages live in the full sunlight throughout the year, and breathe a pure air; they are vigorous, singularly erect in carriage, and accustomed, especially the young women, to carry large quantities of grass and other heavy weights on the head. Their diet principally consists of milk and its products, with polenta, a porridge made of Indian corn-meal—not the starch known as corn-flour in England, but the entire meal, comprising its due proportion of gluten. In the season they also have an abundance of grapes, figs, and chestnuts. Among these people, we rarely see a case of goitre, no cretinism, and, as I have remarked, little *dental* disease.

A sad contrast is presented by the inhabitants of two great valleys lying respectively north and south of the dividing chain of the Alps, and who are of the same races as those inhabiting the mountain slopes.

In the Val d'Aosta, and in the Rhone valley, the people are mainly fed on a similar diet to that of the mountaineers, with the addition of some less simple food, and a greater consumption of wine; but their deep valleys are overshadowed on the southern side by high mountains, which almost entirely shut out the sun in winter-time; these valleys have always been noted for the presence of goitre and cretinism, and precisely in these localities, I found disease of the teeth a common complaint.

Thus we see that general hygienic conditions are often as much concerned in the growth of healthy teeth as food, and the people living in marshy districts present a great contrast to the dwellers in healthy regions; a view of the case which is confirmed by medical friends with regard to several races.

My paper has been discursive, but facts may perhaps be thus better fixed in the memory, and a few deductions may, I think, be fairly drawn from the data. An ample supply of food containing a due proportion of nitrogenous or tissue-forming material is absolutely necessary to the development of healthy teeth. The source from which the proteine is derived is of comparatively little consequence, whether it be raw or cooked flesh, fowl, or fish; milk or its products; the cereals, or fruits or roots, provided only that a sufficient quantity of the element is present; but some modification of the diet is required to suit various climates.

It has usually been supposed that an exclusively vegetable diet is the best adapted to hot countries, but we find that the flesh-abjuring Brahmins are more readily affected by the Indian heat than Englishmen ; they are obese, apathetic, and have less power of resisting disease; they are not long lived, and supply few good soldiers to the army; whilst the lower caste of Hindoos largely supplement their amylaceous food with leguminous seeds, milk, and fish, which are subservient to the formation and reparation of animal tissues.

Nevertheless, the people of Northern India, living on wheat and milk alone, are liable, in the proportion of only one-third the amount, to dental caries, as compared with the inhabitants of the plains.

In very cold countries, on the other hand, in addition to flesh-forming food, there is a constantly urgent demand for oils and fats, that the temperature of the body may be maintained at the normal standard when the thermometer indicates a degree of cold far below zero; and the excessive amount of oleaginous food which, could it be taken in a hot climate, would be stored in the system as fat, only serves to keep up the necessarily energetic vital combustion. We have here a remarkable illustration of the power of the human organization in adapting itself to the various regions of the earth, which has no parallel among the lower animals.

A remarkable difference is obesrvable in regard to the different modes of attrition of the teeth. Those tribes who, from careless perparation of their food, allow sand to mix with it, usually grind down all their teeth equally, as in the notable examples of the Egyptians, and the salmon-eating North American Indians.*

When the aliment consists of hard seeds, roots, or tough animal food, the greatest amount of degradation occurs on the surface of the first molars—towards the lingual side in the upper, and the buccal in the lower jaw.

* A singular contrast is presented by the teeth of the Elephant, with respect to the severe attrition which is absolutely necessary to their efficiency. I was informed by a geutleman in the Indian Civil Service that he found the Elephants belonging to his department were losing flesh and strength. On examining the cause, he found that the soft food with which they were supplied in captivity was altogether insufficient for keeping the three tissues of the teeth—enamel, dentine, and cementum—in the uneven degree of attrition necessary for masticating. Consequently the grinding surfaces of these organs were quite flat and smooth.

Recollecting that in a wild state these animals live on branches and leaves of trees, and on roots which they dig out of the sandy soil, the keepers were ordered to mix sand with their food, and the animals soon recovered their health.

Moreover, with those who live on highly nitrogenized food, the exposed pulp becomes gradually and entirely calcified, and the tooth is often worn down to the neck without giving rise to abscess; but when there is defective nourishment, inflammation, abscess, and extensive alveolar absorption are constantly observable.

Dental caries is not exclusively the attendant of civilization, for it often prevails among the feebler savage tribes whilst it is rare among their more powerful neighbors, by whom their cattle are stolen, and their strongest men killed or carried away into slavery; general deterioration of the race ensues, and the dental tissues are involved in the results of defective nutrition.

I hope we shall obtain some light from these investigations upon that oft-repeated and important inquiry, Why are diseases of the teeth more common now in civilized life than they formerly were?

There are, doubtless, multitudes of feeble lives which, in a ruder state of society, would have succumbed in infancy; but which, by careful nurture, and the advance of medical skill, are saved, and the general health is fairly established. We must, however, remember that the teeth, are not like the other tissues, but, as already observed, when once formed have so slight reparative power that they remain persistently defective; whilst the influence of cumulative hereditary tendency too often operates on succeeding generations.

It is to be feared that a large amount of dental disease is originated by overtaxing the active brain of children.

According to the best authorities, the most rapid increase in the growth of the brain takes place before seven years of age; and it must be remembered that the crowns of all the permanent teeth, with the exception of the third molars, are in the course of development simultaneously with this great advance in the size of the brain. May we not therefore reasonably suppose that through the diminished vitality consequent upon this diversion of the formative energy from the teeth, by premature mental exertion, these organs necessarily become degenerated; and that this circumstance constitutes one great difference between the teeth of the intellectual and those of the uncultivated families of mankind?

It has often been remarked of men who have become eminent for their learning, that they were idle scholars in boyhood; and a celebrated divine has recorded that his father never praised him up to the age of twelve, for anything but his ability to roll great stones, which his elder and more studious brothers could scarcely move; yet, when once he applied himself to study, he made such rapid progress that he became the only member of the family who achieved eminence. I wish it were possible to attain the statistics of the condition of the teeth in these cases, where the brain was not set to hard work until the teeth had obtained their due development. My attention has long been directed to this point, and I feel satisfied that I have traced the existence of disease in the teeth, in many instances, to the prejudicial influence of premature intellectual effort.

It is a recognized fact among ethnologists, that civilization tends to diminish the size of the facial bones. The savage eats his tough or hard food either raw or imperfectly cooked, and an excessive development of the maxillæ and of the zygomatic arch is thereby induced; whereas in civilized life the well-cooked food demands less effort in mastication, and the facial bones become less expanded and robust.

As the relative size of the teeth does not appear to be in a corresponding degree affected, we may thus in many instances account for the want of proportion between the teeth and their sockets, which is not only the cause of irregularity, but also a fruitful source of caries on the approximal surfaces of the teeth.

A reference to the teeth of savage races will show that irregularity is as rare, as destructive attrition is common, among them; whilst these conditions are precisely reversed with respect to the teeth of highly civilized peoples.

I have noticed a striking fact with regard to an uneven structure of the enamel with which we are only too familiar—its irregular deposition on the incisors, cuspidati, and first molars, popularly known as honeycombed teeth; whilst upon the premolars and the second and third true molars this tissue is perfectly developed.

Among the many hundreds of skulls, ancient and modern, which

I have examined, I have met with but two examples of this defect; one of these occurred in the skull of a West-Indian negro, the other in that of a Sandwich Islander, and as the former was living under English, the latter probably under American influences, I think we cannot be mistaken in attributing these exceptional cases to some cause connected with the practices of civilized people, which probably may be the administration of mercury in childhood. I believe that the present generation of adults are, in many cases, suffering from the injury inflicted on their teeth by mercurial medicines administered in infancy; and this, not so much by medical authority as by the treatment of injudicious mothers, who thirty years ago considered calomel and grey powder very innocent domestic remedies; and I am persuaded that I have in many instances traced the connection between such medicines and the dental defect.

Happily for the rising generation these maternal experiments are now generally made with doses as harmless as they are minute.

The adulteration of bread with alum* is, I strongly suspect, a source of much evil, not only in regard to the inferior quality of the flour which is thus made into saleable bread, but to the direct influence of this bi-sulphate upon the enamel. On this subject I have some experiments in progress, which may possibly throw light upon the question. The introduction of this practice is of no recent date, as I unexpectedly met with a complaint respecting it, in one of Smallett's works published exactly a century ago.

We may learn from the circumstance that the rice-eating people of India are obliged to compensate for the poorness of the grain in flesh-forming elements, by adding pulse or some animal food to their diet, that the practice of feeding children with starch, whether it be derived from wheat, arrowroot, maize, or other sources, is, on the principle "ex nihilo nihil fit," incapable

* On visiting an extensive alum manufactory some years since, I inquired if they disposed of the product to the dyers as a mordant; but to my astonishment was told, that the whole of the alum was sold to the millers, excepting a small quantity to bakers, and I saw a large number of sacks full of alum, granulated to the size of pearl-barley, the more readily to be mixed with wheat before grinding. It is, moreover, to be feared that the baker sometimes ignores this, and follows out his own traditional practice by adding a second dose of alum.

of resulting in the formation of healthy tissues, unless abundance of milk or other food rich in nitrogenous principles be added to the diet; and that the entire meal of wheat, deprived of only the coarsest bran, is a far more nutritious food.

It may be a fanciful association of ideas, but I cannot help endeavoring to explain that remarkable tendency to decay in the teeth of children born of English parents in India—whatever care is bestowed on their diet and regimen—by the fact that the more vigorous tribes, who were the ancestors of the present people of Britain, were of northern origin.

It is just possible that the mysterious law which impels the swallow, the nightingale, and other birds inhabiting the north of Africa, to rear their young in our cooler climate, whilst the flocks of wild fowl who spend their winter on our shores and moors, are summoned by the same irresistible instinct to hatch their broods in a region approaching the Arctic circle—may help us to an explanation, and that the tonic influence of our climate is necessary for the development of healthy tissues in children of the British race.

I should not have felt justified in devoting my attention to this subject on so extensive a scale, as a mere question of scientific interest, but I cannot help entertaining the hope that we may derive some practical results from the investigation. Meanwhile, it would be very desirable that accurate statistics should be obtained, in various parts of the United Kingdom, which would enable us to see the relative tendency to disease of the teeth among rural and urban populations, and what relations it may bear to their intellectual advancement and occupations, their food and sanitary conditions.

A comparison between the classes living mainly on wheat, oatmeal, and potatoes would be especially valuable. With this object, it would be very instructive, wherever practicable, to examine the teeth of all the occupants of large schools, factories, workhouses, and other establishments in town and country districts. Elaborate data have already been published on matters relating to general health, and Mr. Tomes has supplied us with very valuable tables, exhibiting the relative tendency of different classes of teeth to decay; but, as far as I am aware, the percentage of individuals subject to dental caries in England has yet to be ascertained. The organization of this society is especially favorable to the prosecution of such research, and I trust that many members will select and work out some particular line of observation, and that the members of our sister society in Scotland will also use their efforts in a similar direction.

I cannot help feeling hopeful with regard to the future; and I trust that due attention to the diet, and appropriate mental training of children, with the constant advance made in the treatment of dental diseases, will be followed by beneficial results to succeeding generations.

In conclusion, I must record my deep sense of obligation to Professor Rolleston, Dr. Davis, and Dr. Thurnam, also to Canon Greenwell; and to Professor Flower for the facilities he has so often afforded me for pursuing my investigations in the Hunterian Museum at unseasonably early hours. My acknowledgments are due to Dr. Birch, to Mr. Newton, and Mr. Franks, of the British Museum; and to Mr. Grenfell and Mr. Hamilton, who have so kindly aided me in consulting works in the library of that institution.

I am much indebted to the medical authorities at Netley, and to the curators of the various hospitals and other museums which I have visited, for the uniform courtesy I have experienced.—*Trans. Odonto. Soc., Great Britain.*

